# Indicated Web-Based Prevention for Women With Anorexia Nervosa Symptoms: Randomized Controlled Efficacy Trial

**DOI:** 10.2196/35947

**Published:** 2022-06-02

**Authors:** Corinna Jacobi, Bianka Vollert, Kristian Hütter, Paula von Bloh, Nadine Eiterich, Dennis Görlich, C Barr Taylor

**Affiliations:** 1 Department of Clinical Psychology and Psychotherapy Faculty of Psychology Technische Universität Dresden Dresden Germany; 2 Institute of Biostatistics and Clinical Research Westfälische Wilhelms-Universität Münster Münster Germany; 3 Department of Psychiatry Stanford University School of Medicine Stanford, CA United States

**Keywords:** anorexia nervosa, internet, indicated prevention

## Abstract

**Background:**

Although preventive interventions for eating disorders in general have shown promise, interventions specifically targeting individuals at risk for anorexia nervosa (AN) are lacking.

**Objective:**

The aim of this study was to determine the efficacy of a guided, indicated web-based prevention program for women at risk for AN.

**Methods:**

We conducted a randomized controlled efficacy trial for women at risk for AN. Assessments were carried out at baseline (before the intervention), after the intervention (10 weeks after baseline), and at 6- and 12-month follow-ups (FUs). A total of 168 women with low body weight (17.5 kg/m^2^≤BMI≤19 kg/m^2^) and high weight concerns or with normal body weight (19 kg/m^2^<BMI≤25 kg/m^2^), high weight concerns, and high restrained eating were recruited from 3 German universities as well as on the web and randomized to Student Bodies-AN (SB-AN; intervention group [IG]) or a wait-list control group (CG). The exclusion criteria were current Diagnostic and Statistical Manual of Mental Disorders, Fourth Edition–based full-syndrome eating disorders and serious medical or mental problems. The interventions were a cognitive-behavioral guided web-based prevention program (SB-AN) over 10 weeks (IG) and a wait-list CG. The primary outcomes were clinically significant changes in disordered eating attitudes and behaviors and change in BMI at 12-month FU in the group of participants who were underweight. The secondary outcomes were new onset of eating disorders, symptoms of disordered eating, and associated psychopathology.

**Results:**

Data were available for 81.5% (137/168) of the women after the intervention and for 69% (116/168) of the women at 12-month FU. At 12-month FU, the IG participants showed larger decreases in Eating Disorder Examination total scores (38/48, 79% vs 33/58, 57%) than the CG participants and the IG participants who were underweight also showed larger clinically relevant increases in BMI (15/31, 49% vs 10/32, 32%) than the CG participants, but these differences were not significant. In addition, after the intervention and at 12-month FU, we found a significant increase in continuously measured BMI for the participants who were underweight and significant improvements in disordered eating attitudes and behaviors (eg, restrained eating as well as weight and shape concerns). At all time points, the rates of new-onset eating disorder cases were (nonsignificantly) lower in the IG than in the CG and the reductions in Diagnostic and Statistical Manual of Mental Disorders, Fifth Edition–based eating disorder syndromes were (nonsignificantly) higher in the IG than in the CG.

**Conclusions:**

SB-AN is the first preventive intervention shown to significantly reduce specific risk factors for, and symptoms of, AN and shows promise for reducing full-syndrome AN onset.

**Trial Registration:**

ISRCTN Registry ISRCTN70380261; https://www.isrctn.com/ISRCTN70380261

## Introduction

### Background

Anorexia nervosa (AN) is a serious condition, often accompanied by severe medical complications and high psychiatric comorbidity [1]. Mortality rates for AN are higher than for any other psychiatric disorder [2,3]. Evidence from controlled treatment trials for AN is limited compared with trials for other eating disorders (EDs), with no specific treatment for older adolescents or adults demonstrating clear superiority over nonspecific treatment [4,5]. Studies addressing long-term outcomes of AN have also demonstrated a rather poor outcome for at least a third to half of the patients [3,6]. Finally, patients with AN also have significantly impaired health-related quality of life and AN is associated with increased health care use and health care costs [7-9]. Given the seriousness and often chronic course of the disorder, early preventive interventions are of crucial importance. These interventions should target modifiable potent risk factors to reduce the onset of the disorder and mitigate core symptoms of the disorder before the onset, thus lowering risk for AN onset. However, although a number of longitudinally assessed risk factors for EDs in general have been confirmed, knowledge regarding specific risk factors for AN is still very scarce [10,11].

Several previous reviews and meta-analyses have examined the efficacy of universal targeted or indicated prevention programs for EDs delivered face-to-face [12-16] or over the internet [12,17-19]. Overall, these reviews found evidence that preventive interventions can reduce potent risk factors for, and symptoms of, EDs, with mostly small to moderate effect sizes. A few individual studies [20-23] also found evidence that preventive interventions can reduce new onset of (mostly) bulimia nervosa (BN) or binge eating–type EDs. However, because specific risk factors for specific ED diagnoses have not been replicated, participants in studies with targeted or indicated programs are usually selected based on general modifiable potent risk factors for ED, such as weight concern, shape concern, or body dissatisfaction. These interventions are not specifically directed at individuals at risk for specific ED diagnoses such as AN. Only recently, in an amalgam of 3 previous prevention trials, Stice et al [24] identified some risk factors with unique predictive effects for ED diagnoses. In the study, low BMI and dieting were found to specifically predict onset of subthreshold or threshold AN. However, based on (21 out of 26) prevention trials included in 2 meta-analyses, the mean BMI of young adult participants was 23.3 (SD 2.8; range 21.6-24.8) kg/m^2^ [15] and 23.5 (SD 0.9; range 21.9-25.5) kg/m^2^ [12]; none of the studies had included lower body weight to determine risk status as the selection criterion. Consequently, adult participants with a lower BMI (ie, BMI<21 kg/m^2^) who may be specifically at risk for AN were not included in these programs.

The question of which variables might moderate intervention effects for specific symptoms or diagnoses of EDs has also hardly been addressed by meta-analyses [19] and individual studies. Of the few studies, 1 found the largest effects of a web-based prevention program on onset of subclinical BN and binge ED (BED) for participants with higher BMI and higher levels of compensatory behaviors at baseline [22]. A second study found lowest intervention effects on abstinence of binge eating, compensatory behaviors, and restrictive eating after the intervention for individuals with purely restrictive eating at baseline [25].

### Prior Work

As part of a pilot study, we specifically designed a web-based indicated preventive intervention (*Student Bodies-AN* [SB-AN]) for this risk group and assessed its feasibility, acceptance, and effectiveness in a pilot study with 36 women, including those with low BMI (<19 kg/m^2^) and higher restrained eating. Overall, the pilot study showed that recruitment of participants at risk for AN with low body weight and high restrained eating is feasible and shows promise. We found significant pre–post reductions in common risk factors for EDs (eg, weight concern) with medium to large effects, as well as specific effects for the underweight subgroup in terms of reductions in restrained eating and an increase in BMI [26].

### Goal of This Study

The major objective of this study was to determine the efficacy of this web-based intervention for women at risk for AN in reducing core risk factors; early symptoms; or syndrome progression of pre-existing, or onset of newly emerging subclinical syndromes of AN compared with a wait-list control group (CG). We hypothesized that the intervention group (IG) participants would show greater improvements in attitudes and symptoms that are more specific for AN, that is, low BMI, and in general ED risk factors such as weight concern and shape concern. In addition, we expected that the participating women would show significantly fewer subclinical ED syndromes at 12-month follow-up (FU).

## Methods

### Study Design and Participants

We conducted a randomized controlled efficacy trial in women at risk for AN. Participants were screened, recruited, and assessed between September 2013 and November 2015 through different faculties at 3 German universities (Dresden, Leipzig, and Halle) and other educational institutions in Dresden through announcements in local media, health insurance membership magazines, and social media (eg, Facebook) as well as through flyer distribution. To ensure a high-enough rate of women with subclinical AN, the study was also announced to women seeking information or help at a secondary advisory center for EDs (ANAD eV, Munich).

We included women aged >18 years with high weight concerns (Weight Concerns Scale [WCS] score>42) and lower body weight (17.5 kg/m^2^≤BMI≤21 kg/m^2^), or with normal body weight (21 kg/m^2^<BMI≤25 kg/m^2^), high weight concerns, and high restrained eating (Eating Disorder Examination [EDE] Questionnaire Restraint score≥2.6, which was >1 SD above the mean of healthy controls [23]). The exclusion criteria were a current Diagnostic and Statistical Manual of Mental Disorders, Fourth Edition (DSM-IV)–based full-syndrome AN, BN, or BED; serious medical or mental problems such as current substance abuse, acute or chronic organic or schizophrenic psychosis, and severe suicidal ideation or behavior; and no internet access.

After the screening, the assessment points were as follows: before the intervention (baseline), midintervention point, after the intervention (10 weeks after baseline), and 6- and 12-month FUs. Quality control methods comprised case report forms, independent data management, on-site monitoring, and documentation of adverse and severe adverse events. The Koordinierungszentrum für Klinische Studien (KKS; Coordination Center for Clinical Trials), Dresden, was responsible for setting up a database according to International Council for Harmonisation Good Clinical Practice requirements, using the software MACRO 4.0 (Microsoft). To ensure data quality, validity and consistency checks were programmed for data entry and regularly checked by a research assistant.

### Ethics Approval

The study was approved by the local ethics committee of Technische Universität Dresden, Germany (EK264082012). Written informed consent was obtained from all participants. The study was conducted according to the Declaration of Helsinki and Good Clinical Practice principles.

### Patient Involvement

Patients with a full-syndrome ED at baseline were excluded from the study. Study participants were not involved in the research question, design of the study, development of outcome measures, or recruitment. However, participant feedback on the intervention included in the pilot study led to some content-related and technical revisions of the intervention (eg, improvement in the dashboard function and the technical usability of the platform, revision of instructions for the symptom checklist and self-monitoring diary, and inclusion of a booster session 2 months after the end of the intervention).

### Randomization and Masking

Concealed randomization was carried out centrally in a ratio of 1:1 by an independent clinical trials center (KKS) after participants had been enrolled in the study and had given informed consent. The randomization was stratified by weight group (underweight, 17.5 kg/m^2^≤BMI≤19 kg/m^2^; low weight, 19 kg/m^2^<BMI≤21 kg/m^2^; normal weight, BMI>21 kg/m^2^) at baseline. A block randomization with random block sizes was used. The KKS also carried out data monitoring and statistical analyses for the main outcomes. The assessors (Anne Buchholz, Sarah Bunzel, Silke Elsäßer, Melanie Hassler, Sabrina Irrgang, Gerda Keil, Francie Kriegel, Franziska Miksch, Annegret Neubauer, Angelika Schuster, Juliane Thieme, Pia Trübenbach, Anna Wagner, and Monique Zobel) who carried out the postintervention and FU assessments were blind to intervention allocation and were not involved in either the moderation of the intervention or the final data analyses.

### Procedures

Participants were recruited through lectures and seminars from different departments with a high proportion of women (eg, psychology or social sciences) or a large number of students in general (eg, business studies) of 3 German universities (Dresden, Halle, and Leipzig). All female students were invited to participate in a study to improve body image and asked to fill out a short screening questionnaire (either a paper-and-pencil version or a web-based version). In addition, the web-based version of this questionnaire was advertised through posters, university mailing lists, flyers, websites of ED associations, local and nationwide media (eg, Facebook), and health insurance companies. Women who screened positive were subsequently invited to a face-to-face or telephone interview where the study was described in detail and informed consent was obtained. Thereafter, the EDE interview [27,28] was administered to assess a current or past ED and participants received log-in data to access the password-protected web-based platform to fill out baseline self-report questionnaires. If participants met the criteria for a current full-syndrome ED, the research team provided treatment recommendations. Postintervention as well as 6- and 12-month FU assessments also included EDE interviews and self-report questionnaires provided through the web-based platform that hosted the intervention.

Participants were provided individual feedback on current ED risk factors (EDE scores, BMI, and ED symptoms) at baseline and on change in the risk factors at postintervention and FU assessments. At the completion of each interview, participants received €20 (US $21.7).

### The Intervention (SB-AN)

We designed the intervention SB-AN based upon existing targeted web-based cognitive-behavioral versions of the intervention *Student Bodies* [22,25,29], expanding its duration from 8 to 10 weekly sessions. The core goals of these programs are to reduce weight concern as well as shape concern, enhance body image, promote healthy weight regulation, and increase knowledge of the risks associated with EDs and specific ED symptoms; for example, binge eating and compensatory behaviors. The program is supplemented by a web-based asynchronous moderated discussion group. Other elements include a personal journal and a body image journal.

For this study, we made contextual adaptations according to the special needs of the groups with higher restrained eating or lower weight. In anticipation of the noted ambivalence to change in this population, we added elements of motivational interviewing [30] to the first sessions (eg, pros and cons of low weight and restrained eating). We also expanded the psychoeducational content on EDs to increase participants’ awareness of their current eating and exercise behavior as well as body image compared with patients with other EDs. Furthermore, compared with programs addressing non-AN EDs, the program focused more specifically on restrictive eating. Other topics addressed were media literacy, coping with negative emotions, improving social skills, and healthy eating and exercise. The symptom checklist that was integrated into the web-based program was expanded to include frequencies of core ED symptoms (body weight, restrained eating, meals per day, missed meals, reduced meals, avoided foods, objective and subjective episodes of binge eating, episodes of vomiting, laxative abuse, abuse of diuretics or appetite suppressants to control weight, and driven exercise). To normalize their eating behavior and reduce ED symptoms (eg, restrained eating, binge eating, and purging), participants were prompted weekly to fill out the symptom checklist and given individual weekly feedback by the program moderators on their entries in the symptom checklist and other interactive program elements (ie, personal and self-monitoring logs and contributions to the web-based discussion group). The program was moderated by psychology (master’s degree or diploma level) graduate students in training for behavior therapy who were supervised by a licensed clinical psychologist (CJ). The feedback was intended to foster reflection on, and change in, dysfunctional eating and weight-related thoughts and behaviors. Each session of the program took 45 to 90 minutes to be completed. The program’s home page also provided short résumés of the program moderators once participants had logged in to facilitate the credibility of the intervention.

### Wait-list CG

Given that SB-AN is the first prevention program specifically targeting women at risk of AN, no alternative interventions (*treatment as usual*) exist. A wait-list CG therefore seemed to be the first-choice control condition and ethically justifiable to determine the efficacy of the intervention. Participants assigned to the CG were assessed at all interview assessment points and offered to participate in the program after completion of the 12-month FU.

### Outcome Measures

Outcomes were selected to reflect core features of the included risk group and based on preliminary effects found in the pilot study [26]. The primary outcomes were rates (in percentages) of participants with a decrease in EDE interview total score [31] below a score of 1.87 between before the intervention and 12-month FU (reflecting a clinically significant change) and rates (in percentages) of participants who were underweight with a BMI increase of at least 0.8 kg/m^2^ between screening and 12-month FU.

The secondary outcomes were continuously measured BMI of participant who were underweight, disordered eating attitudes and behaviors, numbers of subjective and objective binge eating episodes (for the binge eating subgroup), and rates of participants fulfilling criteria for onset of a full-syndrome and subclinical ED.

Disordered eating attitudes and behaviors were assessed by the WCS [32], the EDE (total score; subscales: Weight Concern, Shape Concern, Eating Concern, and Restraint; and numbers of objective and subjective binge eating episodes [27]), and the Eating Disorder Inventory-2 (EDI-2) subscales Drive for Thinness and Body Dissatisfaction [33]. For all measures, good psychometric properties have been reported for both the original and the German-validated versions [28,31,32,34-37]. Additional measures covered associated psychopathology such as general psychopathology (Brief Symptom Inventory [38]) and depression (Beck Depression Inventory [39]), as well as a knowledge test concerning program content. Good psychometric properties have been reported for all these measures for both the original versions and the German adaptations [40,41]. To assess clinical impairment due to ED psychopathology we used our own translation of the Clinical Impairment Assessment [42,43].

After the study had begun, DSM, Fifth Edition (DSM-5) [44] was published, which included slight changes to some of the diagnostic criteria. The new classification system loosens the criteria for some EDs, resulting in individuals who were not diagnosed as being ED cases with DSM-IV now becoming ED cases with DSM-5. We decided to adopt DSM-5 criteria for all baseline and FU assessments. This resulted in 9 individuals becoming full-syndrome AN cases at baseline, which allowed us to determine the treatment effects of the intervention in reducing symptoms in this group. To determine the preventive effect of the intervention, we excluded all DSM-5 cases at baseline and only examined individuals who became cases according to the new criteria.

In addition, participants who met all criteria in accordance with the following definitions were considered to be cases of subclinical ED (Eating Disorder Not Otherwise Specified and Other Specified Feeding or Eating Disorder [44]) at baseline or subsequent assessment points: subclinical AN: (1) 18.5 kg/m^2^>BMI<19.2 kg/m^2^), (2) fear of weight gain in the past 3 months (DSM-5 criterion B), and (3) either undue influence of body weight or shape on self-esteem or feeling fat on more than half of the days in the past 3 months (DSM-5 criterion C) or (1) BMI<18.5 kg/m^2^ and (2) DSM-5 criterion B or C. Subclinical BN: All DSM-5 criteria for BN are met except undue influence of body weight and shape on self-esteem (DSM-5 criterion D). Subclinical BED: All DSM-5 criteria are met except marked distress regarding binge eating (DSM-5 criterion C).

All interviews were conducted by assessors not involved in intervention moderation and data analyses. They completed a 2-day workshop during which they were trained on the EDE interview assessments, on the use of the database for the assessments, and on providing feedback regarding ED psychopathology to participants. Feedback to participants was recorded, and the assessors were supervised by graduate students regarding the quality of the feedback provided. Over the course of the trial, interviewer trainings were repeated for new interviewers.

BMI was measured using a portable stadiometer measuring height to the nearest millimeter and a digital scale measuring weight to the nearest 0.1 kg. In case of telephone interviews, BMI was calculated based on self-reported height and weight (obtained during the interview). All primary and secondary outcomes were assessed at all 4 assessment points. After the intervention as well as at 6- and 12-month FUs we also assessed the use of any additional inpatient, outpatient, or day-patient treatment that patients had received since the start of their participation in the SB-AN study.

### Sample Size

We based sample size calculations on the assumption that 50% of the IG and 20% of the CG would show a decrease in EDE total score below the critical value of 1.87. Applying the Fisher exact test based on a power of at least 80%, the required sample size for the analysis would be 44 participants per group or a total of 88 participants.

For the group of participants who were underweight, we assumed that a rate of 50% of the participants in the IG succeeding in increasing their BMI between before the intervention and 12-month FU by at least 0.8 kg/m^2^ compared with 5% of the CG participants represented a clinically significant difference. To detect this difference, 34 (17 in each group) participants would be required in the group of participants who were underweight (BMI between 17.5 kg/m^2^ and 19 kg/m^2^; based on the Fisher exact test with α=.05 and power of 80%).

Assuming similar rates of returned screens (74%), women who screen positive (13.9% of the screened women), and eligible women (31.9% of the screened positives) as in the pilot study, we would have to administer 8273 screening questionnaires to obtain 6122 (74%) returned screens, of which 851 (13.9%; n=448, 7.32%, needed) would be women who screen positive. Of these 448 women, 143 (31.92%) would be eligible.

Conservatively estimating an attrition rate of 45% until 12-month FU (15% of the participants after the intervention and another 15% at each of the FU assessments), 88 participants would provide sufficient data for the analyses.

### Statistical Analyses

Study data are described using absolute and relative frequencies for categorical variables. Continuous outcomes are described using means and SDs. All analyses of primary and secondary outcomes were conducted as intention-to-treat analyses. We analyzed the study data according to the study protocol and as outlined in the study registry.

### Primary Outcomes

Originally, we planned to use the Fisher exact test to compare the 12-month FU rates of participants in the IG and CG fulfilling the primary outcome variables (percentage of participants who were underweight with a BMI increase of at least 0.8 kg/m^2^ and percentage of participants with EDE total score of no more than 1.87 with baseline score above 1.87) at a significance level of *P*=.05 for the primary analysis. In this analysis, missing data were imputed using the last observation carried forward (LOCF) method.

As LOCF makes assumptions that can yield biased results and is no longer considered the best method to impute missing data, we also used the more robust method of generalized linear mixed models (GLMMs) [45]. Here, the full available data from each participant at all assessments were considered in the analyses using the maximum likelihood method. We entered fixed effects for randomized group, time, and the group×time interaction. Random intercepts, allowing us to model repeated measurements and to account for heterogeneity in outcomes across individuals, were fitted. To model longitudinal data appropriately, we used a covariance matrix with a first-order autoregressive structure. The primary outcome variables were modeled with a binomial distribution and a logit link. To test differences in binary outcomes between the IG and the CG, Wald test *P* values were calculated.

### Secondary Outcomes

GLMMs were used to analyze continuous secondary outcomes as well. Data from each participant at all assessments (baseline, midintervention point [except for EDE scales, BMI, and binge eating episodes], after the intervention, and FUs) were considered in the analyses. We entered fixed effects for randomized group, time, and the group×time interaction and calculated suitable contrasts to test the changes from baseline to all subsequent time points between the groups. Random intercepts were fitted, and we used a covariance matrix with a first-order autoregressive structure. For skewed outcome variables, we used the best fitting distribution (eg, *γ*) for the model. The canonical link function was chosen for all models.

Changes in dichotomous secondary outcomes (diagnoses) between the groups were analyzed using Fisher exact tests for each assessment point separately.

We also analyzed the onset of a new DSM-5–based diagnosis by means of a time-to-event analysis. Observation times were determined as days between baseline and date of onset or last observation. Participants without an onset were censored in the analysis at their last observation date. Cumulative incidence curves were calculated as 1 minus the Kaplan-Meier estimate. Numbers at risk are given along with the incidence curves. Groups were compared using the log-rank test.

### Effect Sizes

We constructed Cohen *d–*like effect sizes from the GLMMs using the known relation between *t* statistics and effect sizes [46]. The resulting effect size can be interpreted similar to common Cohen *d* because it quantifies the effect as standardized estimated mean differences. For binary outcomes, odds ratios were reported as effect size. In addition, we calculated the number needed to treat (NNT) or number needed to harm based on the absolute risk reduction (ARR) [47]. For the time-to-event analysis, hazard ratios and 95% CIs were calculated using a Cox proportional hazards model.

No multiple-testing correction was applied for the analyses of the secondary outcomes because these results are considered explorative. Analyses were performed using SPSS software (version 24.0; IBM Corp) and, for the GLMMs, SAS software (version 9.4 TS1M3, SAS/STAT 14.1; SAS Institute Inc).

## Results

### Recruitment

**Figure 1:**
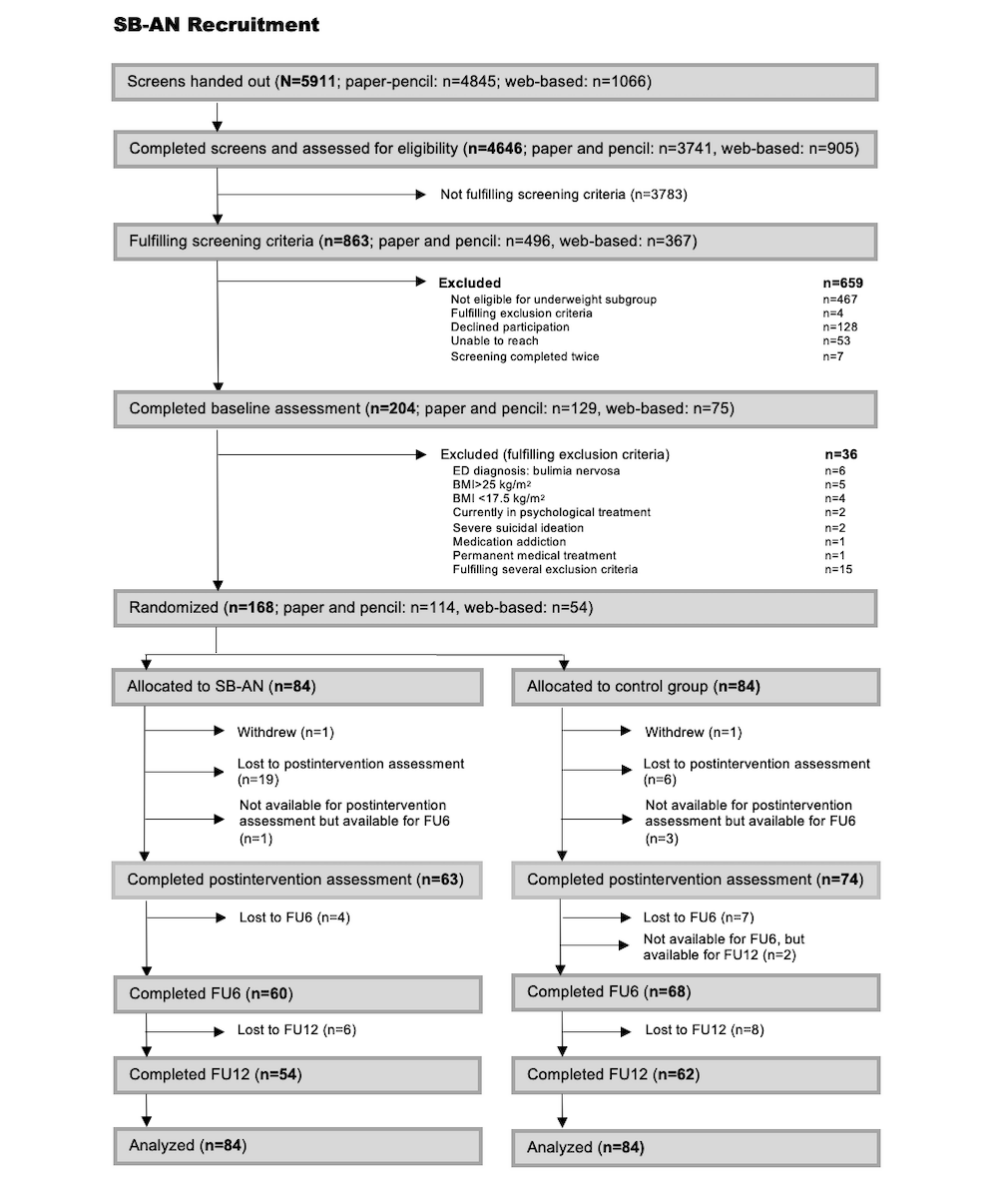
CONSORT (Consolidated Standards of Reporting Trials) flow of participants. ED: eating disorder; FU6: 6-month follow-up; FU12: 12-month follow-up; SB-AN: Student Bodies-Anorexia Nervosa.

Between September 2013 and July 2014, a total of 4646 women were screened for inclusion (n=3741, 80.5%, based on paper-pencil screening and n=905, 19.5%, based on web-based screening); 333 (7.17%) were invited to the preintervention interviews and 168 (3.62%) were randomized to the SB-AN or wait-list control condition. As the recruitment of participants who were underweight took longer in this trial than in the pilot study, we continued our recruitment beyond the originally calculated sample size to ensure a large enough subgroup of participants who were underweight for the main analyses. This resulted in an overall almost doubled sample size of randomized participants. At the end of the intervention period, of the 168 participants, 137 (81.5%) had completed assessments, and at 12-month FU, 64% (54/84) of the participants in the IG and 74% (62/84) of the participants in the CG had completed assessments, resulting in an overall dropout rate of 31% (52/168; 30/84, 36%, and 22/84, 26%, respectively; *P*=.24; Figure 1). Overall, most (114/168, 67.9%) of the EDE assessments of the randomized participants were carried out in person.

The participating women were on average aged 23.3 (SD 3.77) years. Most (140/168, 83.3%) were students from the eastern parts of Germany (Saxony). The average BMI of the sample was 20.08 (SD 1.72) kg/m^2^, with 37.5% (63/168) in the lower or underweight BMI range (17.5 kg/m^2^≤BMI≤19 kg/m^2^). All women showed on average high restrained eating (based on the EDE Restraint subscale) and high weight concerns (based on the WCS). In addition, 12.5% (21/168) of the women reported objective binge eating episodes and vomiting to control weight up to 20 times in the past 4 weeks whereas 11.3% (19/168) engaged in abuse of laxatives or diuretics to control weight up to 32 times in the past 4 weeks. On the basis of the DSM-5 criteria [45] that were published over the course of the study, 5.4% (9/168) of the women (3/84, 4%, in the IG and 6/84, 7%, in the CG) met the criteria for full-syndrome AN. In addition, 7.1% (12/168) of the women (7/84, 8%, in the IG and 5/84, 6%, in the CG) met the criteria for subthreshold AN, whereas 1.2% (2/168) of the women (1/84, 1.2%, in the IG and 1/84, 1.2%, in the CG) met the criteria for subthreshold BN. Table 1 summarizes baseline sociodemographic characteristics and Table 2 shows the baseline clinical scores of all participants.

**Table 1:** Baseline sociodemographic and clinical characteristics of participants (N=168).

			All participants		Intervention group (n=84)			Control group (n=84)	
Age (years), mean (SD)			23.23 (3.77)		22.93 (3.56)			23.53 (3.97)	
**Education level, n (%)**									
	University degree			48 (28.5)		22 (26.2)			26 (30.9)
	Professional qualification			7 (4.2)		4 (4.8)			3 (3.6)
	High school diploma			108 (64.3)		56 (66.7)			52 (61.9)
	Secondary school certificate			5 (3)		2 (2.4)			3 (3.6)
**Occupation, n (%)**									
	Employee	20 (11.9)					8 (9.5)	12 (14.3)	
	Student	140 (83.3)					73 (86.9)	67 (79.8)	
	Apprentice	2 (1.2)					1 (1.2)	1 (1.2)	
	Other	6 (3.6)					2 (2.4)	4 (4.8)	

**Table 2:** Baseline clinical characteristics of participants (N=168).

	All participants		Intervention group (n=84)			Control group (n=84)	
	Value, n (%)	Value, mean (SD)	Value, n (%)	Value, mean (SD)	Value, n (%)		Value, mean (SD)
BMI (T0^a^)	168 (100)	20.08 (1.72)	84 (100)	20.14 (1.76)	84 (100)		20.02 (1.69)
BMI (T1^b^)	168 (100)	20.46 (1.80)	84 (100)	20.51 (1.82)	84 (100)		20.41 (1.78)
BMI (UW^c^-T0)	63 (37.5)	18.40 (0.44)	31 (36.9)	18.43 (0.45)	32 (38.1)		18.38 (0.43)
BMI (UW-T1)	41 (24.4)	18.39 (0.37)	20 (23.8)	18.43 (0.39)	21 (25)		18.35 (0.35)
Binges, objective	14 (8.3)	5.86 (5.22)	6 (7.1)	5.50 (2.59)	8 (9.5)		6.13 (6.75)
Binges, subjective	43 (25.6)	7.16 (5.38)	21 (25)	7.43 (5.54)	22 (26.2)		6.91 (5.34)
Binges, objective+subjective	54 (32.1)	7.22 (5.43)	26 (31)	7.27 (5.39)	28 (33)		7.18 (5.58)
Purging	23 (13.7)	6.78 (6.54)	10 (11.9)	9.60 (9.11)	13 (15.5)		4.62 (2.14)
ED^d^ diagnosis	23 (13.7)	N/A^e^	11 (13.1)	N/A	12 (14.3)		N/A
EDE^f^ total	168 (100)	2.35 (1.07)	84 (100)	2.21 (1.00)	84 (100)		2.49 (1.13)
EDE RS^g^	168 (100)	2.54 (1.30)	84 (100)	2.46 (1.29)	84 (100)		2.62 (1.32)
EDE EC^h^	168 (100)	1.35 (1.11)	84 (100)	1.16 (1.01)	84 (100)		1.53 (1.17)
EDE SC^i^	168 (100)	3.02 (1.28)	84 (100)	2.90 (1.23)	84 (100)		3.15 (1.33)
EDE WC^j^	168 (100)	2.48 (1.37)	84 (100)	2.30 (1.34)	84 (100)		2.67 (1.39)
EDI-2^k^ BD^l^	168 (100)	38.29 (9.57)	84 (100)	36.98 (9.87)	84 (100)		39.61 (9.14)
EDI-2 BUL^m^	168 (100)	13.11 (5.69)	84 (100)	12.76 (5.06)	84 (100)		13.45 (6.27)
EDI-2 DFT^n^	168 (100)	27.99 (7.86)	84 (100)	26.92 (7.69)	84 (100)		29.06 (7.94)
WCS^o^	168 (100)	59.50 (17.82)	84 (100)	55.85 (16.18)	84 (100)		63.16 (18.71)
BDI^p^	168 (100)	12.50 (8.54)	84 (100)	11.74 (8.62)	84 (100)		13.26 (8.44)
BSI^q^	168 (100)	0.71 (0.55)	84 (100)	0.65 (0.56)	84 (100)		0.78 (0.53)
CIA^r^ total score	168 (100)	12.61 (9.77)	84 (100)	10.98 (8.76)	84 (100)		14.25 (10.48)
Knowledge test	168 (100)	18.07 (2.61)	84 (100)	18.11 (2.57)	84 (100)		18.02 (2.67)

^a^T0: screening.

^b^T1: baseline.

^c^UW: underweight.

^d^ED: eating disorder.

^e^N/A: not applicable.

^f^EDE: Eating Disorder Examination.

^g^RS: Restraint.

^h^EC: Eating Concern.

^i^SC: Shape Concern.

^j^WC: Weight Concern.

^k^EDI-2: Eating Disorder Inventory-2.

^l^BD: Body Dissatisfaction.

^m^BUL: bulimia nervosa.

^n^DFT: Drive for Thinness.

^o^WCS: Weight Concerns Scale.

^p^BDI: Beck Depression Inventory.

^q^BSI: Brief Symptom Inventory.

^r^CIA: Clinical Impairment Assessment.

### Effects of the Intervention on Primary and Secondary Outcomes

The analysis of the primary outcomes in the intention-to-treat sample using the Fisher exact test and the LOCF method revealed no significant differences between the IG and the CG at 12-month FU (EDE criterion, *P*=.99; weight gain criterion, *P*=.99). On the basis of the mixed model analyses of the primary outcomes, 79% (38/48) of the IG participants and 57% (33/58) of the CG participants with baseline EDE total scores above 1.87 showed a decrease in EDE total scores below 1.87 at 12-month FU, but this difference was again not significant (*P*=.19). In addition, 49% (15/31) of the IG participants who were underweight and 32% (10/32) of the CG participants who were underweight showed a BMI increase of at least 0.8 points. This difference was also not significant (*P*=.59; Table 3).

On the basis of mixed model analyses of the secondary outcomes, there was a significant group×time interaction in continuously measured BMI in the underweight subgroup between screening and 12-month FU. Furthermore, we found a significant group×time interaction for EDE Restraint, EDI Drive for Thinness, and EDI Body Dissatisfaction for all participants and for subjective and objective binge eating episodes for the subgroup of participants with symptoms of binge eating at 12-month FU. After the intervention, a significant group×time interaction was found for the EDE total score, EDE Restraint, and EDE Shape Concern; for EDI Drive for Thinness and EDI Body Dissatisfaction; for Weight Concerns; for the Beck Depression Inventory and the Clinical Impairment Assessment total scores; and for the knowledge test for all participants. For all secondary outcomes, the IG participants showed larger reductions (ie, more positive effects) than the CG participants, with effect sizes ranging from medium to large (Multimedia Appendix 1).

**Table 3:** Primary outcomes.

Outcome	Analysis	IG^a^, n (%)	CG^b^, n (%)	*P* value^c^	OR^d^ (95% CI)^e^
BMI increase of at least 0.8 kg/m^2^ in participants who were underweight (IG, N=31; CG, N=32)	LOCF^f^	10 (32)	11 (34)	.99	0.91 (0.32-2.59)
BMI increase of at least 0.8 kg/m^2^ in participants who were underweight (IG, N=31; CG, N=32)	GLMM^g^	15 (49)	10 (32)	.59	2.04 (0.15-28.31)
EDE^h^ total score below 1.87 (participants: IG, N=48; CG, N=58)	LOCF	24 (50)	28 (48)	.99	1.07 (0.50-2.30)
EDE total score below 1.87 (participants: IG, N=48; CG, N=58)	GLMM	38 (79)	33 (57)	.19	2.87 (0.60-13.67)

^a^IG: intervention group.

^b^CG: control group.

^c^*P* values correspond to the Fisher exact test for the last observation carried forward imputation and Wald tests for the generalized linear mixed model.

^d^OR: odds ratio.

^e^Odds ratios and 95% CIs were calculated in a logistic regression model.

^f^LOCF: last observation carried forward.

^g^GLMM: generalized linear mixed model for a binary outcome with logit link estimated with the unimputed data using a fixed effects model with *γ*=group, time, group×time, and a random effect for the repeated measurements. Response rates are marginal estimates shown as percentages.

^h^EDE: Eating Disorder Examination.

### Effects of the Intervention on ED Cases

#### Prevention Effects

To assess the prevention effects of the intervention we compared all available IG and CG participants without any full-syndrome or subthreshold EDs at baseline (145/168, 86.3%) with respect to the newly emerging DSM-5 diagnoses at subsequent assessment points. After the intervention, data from 82.1% (119/145) of the participants were available; at 6-month FU, data from 80% (116/145) were available; and at 12-month FU, data from 72.4% (105/145) were available (Table 4).

After the intervention, among the 56 IG participants, 3 (5%) new-onset subclinical ED cases (n=2, 67%, subclinical AN and n=1, 33%, subclinical BN) emerged. Among the 63 CG participants, 5 (8%) new-onset cases (n=4, 80%, subclinical AN and n=1, 20%, subclinical BN) emerged (Fisher exact test, *P*=.72). At 6-month FU, among the 53 IG participants, 1 (2%) subclinical case of AN was observed, and among the 59 CG participants, 4 (7%) new-onset cases (n=1, 25%, full-syndrome case of AN and n=3, 75%, subclinical cases of AN) were observed (Fisher exact test, *P*=.37). Finally, at 12-month FU, among the IG participants (n=48), no new-onset cases emerged, whereas among the 55 CG participants, 3 (5%) new-onset cases (n=2, 67%, subclinical cases of AN and n=1, 33%, subclinical case of BN) were diagnosed (Fisher exact test, *P*=.13).

**Table 4:** Treatment and prevention effects.

Effect	After the intervention					FU6^a^					FU12^b^			
	IG^c^, n/N (%)	CG^d^, n/N (%)	*P* value^e^	OR^f^ (95% CI)	IG, n/N (%)		CG, n/N (%)	*P* value	OR (95% CI)	IG, n/N (%)		CG, n/N (%)	*P* value	OR (95% CI)
Treatment	3/7 (43)	6/10 (60)	.64	0.50 (0.07-3.55)	1/7 (14)		4/9 (44)	.31	0.21 (0.02-2.52)	0/5 (0)		4/6 (67)	.06^g^	0.0 (0.0-1.1)^g^
Prevention	3/56 (5)	5/63 (8)	.72	0.66 (0.15-2.90)	1/53 (2)		4/59 (7)	.37	0.26 (0.03-2.46)	0/48 (0)		3/55 (5)	.13^g^	0.0 (0.0-1.9)^g^

^a^FU6: 6-month follow-up.

^b^FU12: 12-month follow-up.

^c^IG: intervention group.

^d^CG: control group.

^e^All *P* values are from the Fisher exact test.

^f^OR: odds ratio.

^g^The *P* value and the odds ratio of the 12-month FU treatment and prevention effects were estimated with a 0.5 correction of the underlying frequency table to reach a more stable estimate of the odds ratio in this extreme case of results.

#### Time to First Diagnosis

We also analyzed time to first onset of a newly emerging DSM-5–based full-syndrome or subclinical ED diagnosis, that is, days between baseline and the first FU assessment point where a participant met any of these diagnoses. Participants without any diagnosis at any assessment point were included as censored cases in the analysis with their time from baseline until the last available interview.

**Figure 2:**
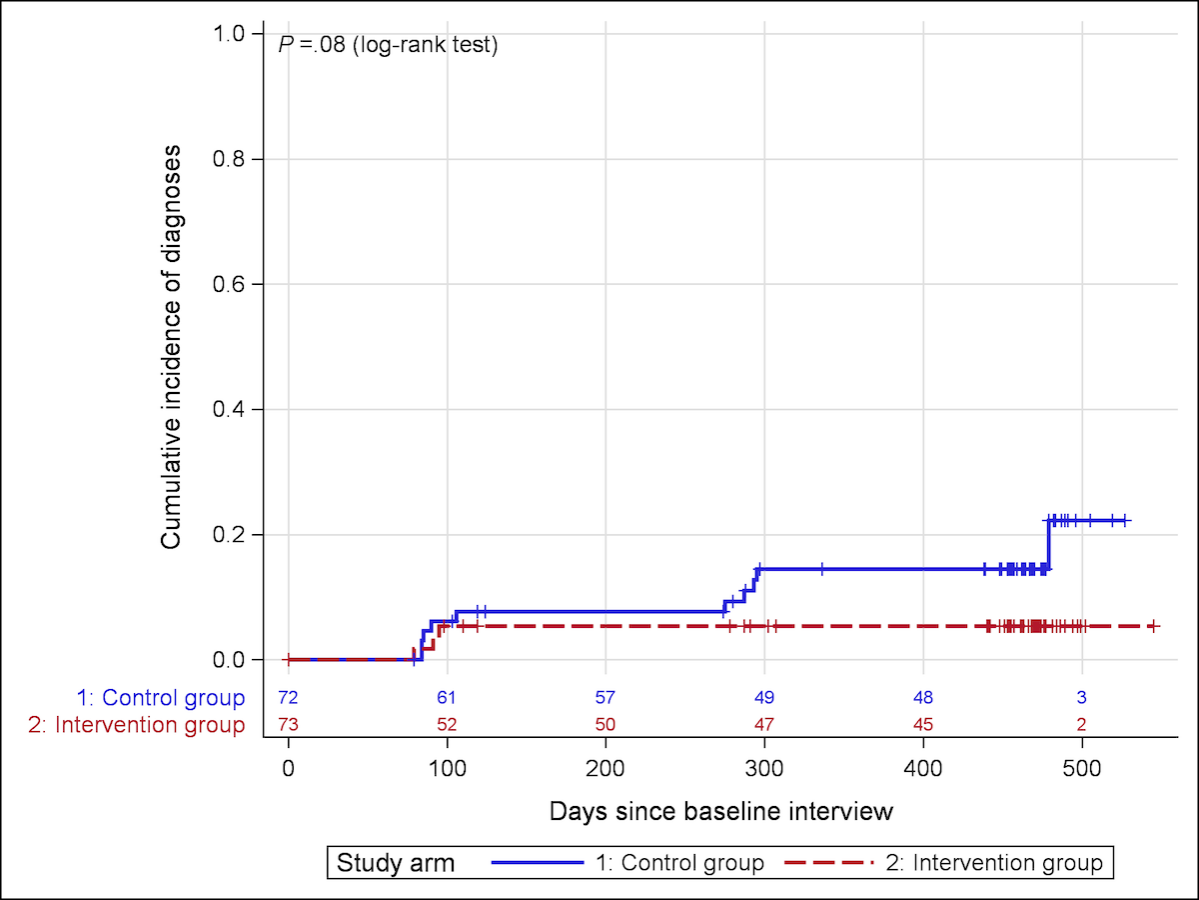
Cumulative incidence curves for new-onset Diagnostic and Statistical Manual of Mental Disorders, Fifth Edition, diagnoses in the intervention and control groups. The respective numbers of participants at risk are provided below the incidence curves.

In the IG, new diagnoses only emerged around the intervention. In the CG, new diagnoses also occurred at 6-month FU and 12-month FU. The estimated 1-year cumulative incidence was 14.6% in the CG and 5.4% in the IG (*P*=.08; hazard ratio 0.335, 95% CI 0.092-1.216; Figure 2).

We calculated the ARR and NNT from the estimated survival curves. The ARR was 0.091 (95% CI 0.07-0.11), which translates into an NNT for the benefit of 11 participants to prevent at least one onset within 12 months after the program ends. The 95% CI of the NNT was 8.97-14.14.

#### Treatment Effects

At baseline, of the 168 participants, 23 (13.7%; IG: n=11, 48%; CG: n=12, 52%) met DSM-5 criteria for full-syndrome and subclinical ED (n=9, 39%, cases of AN; n=12, 52%, cases of subclinical AN; and n=2, 9%, cases of subclinical BN).

After the intervention, of the 168 participants, 17 (10.1%) were available. Of the 10 CG participants, 6 (60%) still met the DSM-5 criteria, whereas of the 7 IG participants, 3 (43%) met the criteria (Fisher exact test, *P*=.64).

At 6-month FU, of the 168 participants, 16 (9.5%) were available. Of the 9 CG participants, 4 (57%) still met the DSM-5 criteria, whereas of the 7 IG participants, 1 (14%) met the criteria (Fisher exact test, *P*=.31). At 12-month FU, none of the IG participants (n=5) met the DSM-5 criteria, whereas of the 6 CG participants, 4 (66%) still met the criteria for full-syndrome and subclinical ED (n=1, 25%, case of AN; n=2, 50%, cases of subclinical AN; and n=1, 25%, case of BN; Fisher exact test, *P*=.06; Table 4).

We also analyzed changes in DSM-5 status for each pairwise transition between 2 assessment points within each study arm using McNemar tests (Table 5). In the IG, a significant proportion of the participants improved over time comparing baseline with 12-month FU and after the intervention with the FU assessment points. For the CG, such a trend could not be shown.

**Table 5:** Pairwise differences on eating disorder diagnoses. Control group results are displayed in the upper right triangle, intervention group results in the lower left triangle.

	Baseline			After the intervention			6-month FU^a^			12-month FU	
	Chi-square (*df*)	*P* value	Chi-square (*df*)		*P* value	Chi-square (*df*)		*P* value	Chi-square (*df*)		*P* value
Baseline	N/A^b^	N/A	0.1 (1)^c^		.74^c^	0.1 (1)^c^		.74^c^	0.2 (1)^c^		.65^c^
After the intervention	0.1 (1)^d^	.71^d^	N/A		N/A	0.1 (1)^c^		.74^c^	1.0 (1)^c^		.32^c^
6-month FU	3.6 (1)^d^	.06^d^	4.0 (1)^d^		.05^d^	N/A		N/A	0 (1)^c^		.99^c^
12-month FU	5.0 (1)^d^	.03^d^	5.0 (1)^d^		.03^d^	1.0 (1)^d^		.32^c^	N/A		N/A

^a^FU: follow-up.

^b^N/A: not applicable.

^c^For control group.

^d^For intervention group.

### Program Adherence

Of the 84 IG participants, 11 (13%) never logged on to the program. Of the remaining 73 women, 53 (72%) opened at least half of the sessions and 47 (64%) accessed at least half of the intervention content. On average, all intervention participants, including those who never logged on to the program, opened 55.4% (SD 40.7; median 62.4%, IQR 87.3%) of the program pages and accessed 6.6 (SD 4.0; median 9%, IQR 8%) of the 10 sessions. Active participants who logged on to the program at least once on average opened 63.8% (SD 37.0%; median 83.3%, IQR 77.39%) of the program pages and accessed 7.6 (SD 3.3; median 10, IQR 6) of the 10 sessions.

### Treatment Seeking

After the intervention, of the 84 CG participants, 1 (1%) reported having resumed treatment after study start. At 6- and 12-month FUs, of the 168 participants, 3 (1.8%) women (n=1, 33%, in the IG and n=2, 67%, in the CG) reported having resumed outpatient treatment for an ED.

## Discussion

### Principal Findings

This is the first study to evaluate the efficacy of an indicated preventive web-based intervention (SB-AN) for young women at risk for AN in reducing risk factors and symptoms of AN as well as syndrome progression of pre-existing, and onset of newly emerging subclinical syndromes of, AN compared with a wait-list CG. The intervention was specifically developed to target early symptoms and potential risk factors for AN that distinguishes SB-AN from other preventive interventions for EDs.

For our primary outcomes we found that the proportion of participants showing reductions in EDE interview total scores at 12-month FU below a score of 1.87 was 22% (79% vs 57%) larger in the IG than in the CG, but this difference was not significant. In addition, larger proportions of IG participants who were underweight showed a BMI increase of at least 0.8 points (49% vs 32%) compared with CG participants who were underweight, but this difference was again not significant. However, medium to large effects of the intervention were seen in several of the secondary outcomes of ED pathology: after the intervention, there were larger reductions in disordered eating attitudes such as restrained eating, shape concern, drive for thinness, body dissatisfaction, weight concern, depression, and clinical impairment in the IG than in the CG. At 12-month FU, the IG still showed larger reductions in restrained eating, drive for thinness, and body dissatisfaction than the CG. The intervention also proved effective in reducing symptoms of disordered eating: subjective and objective binge eating episodes for the subgroup of participants with symptoms of binge eating were significantly lower at 12-month FU, and continuously measured BMI of the subgroup of participants who were underweight was significantly larger in the IG than in the CG. Although fewer IG participants developed DSM-5 new-onset full-syndrome and subclinical EDs than the CG participants, this difference was not significant, probably because of the small numbers of overall cases. In addition, there was a trend for IG participants to develop new-onset cases only in the period around the intervention and not thereafter, whereas CG participants developed new-onset cases over the whole course of the study. Finally, there was also a trend regarding a treatment effect of the intervention, that is, a reduction in DSM-5–based ED diagnoses between baseline and 12-month FU. Fewer IG participants also resumed treatment for their ED during that time. Finally, the NNT at 12-month FU also indicates a benefit of the intervention.

### Strengths and Weaknesses of the Study

Participants in this trial were specifically selected because they were at risk for AN based on either low or lower BMI and clinically elevated restrained eating scores. Stratification for weight group resulted in a mean BMI markedly lower than in other prevention trials for ED [12], with 37.5% (63/168) of the participants falling in the lower-weight to underweight range. The study sample was rather large, and the 12-month FU allowed for assessing the sustainability effects of the intervention. ED diagnoses and AN symptomatology were obtained using a well-validated clinical interview. We used central randomization, conducted the analyses blinded for IG, and controlled for missing data by multiple imputation in the analyses. The intervention itself is easily accessible for participants and likely to be more cost-effective than face-to-face interventions.

However, there were also several limitations. The reach of the intervention [48] was limited: because of our relatively strict inclusion criteria, only 3.62% (168/4646) of the participants who had filled out screens and 19.5% (168/863) of those who had screened positive could be included. Of the 168 participants, 141 (83.9%) were students; hence, the generalizability of the results for more diverse populations remains unclear. Participants could not be blinded as in most other psychological interventions. Although adherence was comparable with other studies that included targeted preventive interventions for ED [49], 13% (11/84) of the randomized participants never logged on to the intervention, and on average, participants used only half to two-thirds of the program. One could assume that higher adherence may have more pronounced intervention effects; however, it may be worthwhile to test the effect of an abbreviated version of the intervention as part of future research.

Attrition was substantial, with rates of 36% (30/84) in the IG and 26% (22/84) in the CG, although again not unusually high compared with other preventive web-based interventions [49]. The selection of dichotomous primary outcomes (EDE total score and BMI differences) was based on uncontrolled effect sizes found in the pilot study [26], which had a much smaller sample size. These uncontrolled effect sizes may not be representative for a larger sample and may have overestimated the true effects. Women in the CG also improved over time on many outcomes, which may indicate that the extensive interviews with feedback on ED symptomatology itself also yielded effects. To control for these potential assessment effects, we would have needed to include a third assessment-only group. Nevertheless, effect sizes for secondary outcomes were all in the medium to large range. Health economic outcomes were not included in the study.

### Comparison With Other Studies

To our knowledge, this is the first study to specifically target young women at risk for AN based on risk factors and early symptoms that may be uniquely predictive for AN onset. Most previous trials that included targeted preventive interventions used weight concern or body dissatisfaction as selection criteria. We found higher intervention effects after the intervention and at FU on ED risk factors (weight concern, shape concern, drive for thinness, dieting, and body dissatisfaction) and bulimic symptomatology compared with previous prevention trials [13,17-19]. The study is unique in promoting weight gain, at least in the lower-weight group. Many prevention studies have either not shown changes in weight [23,29] or targeted weight loss as an outcome [50,51]. This study is also unique in suggesting a preventive effect of late-onset ED cases in women at risk for AN. Only very few face-to-face and web-based ED prevention programs have significantly reduced ED onset [20-23,29,52]. With the exception of 1 case of AN in the study by Taylor et al [23], cases in these studies were subthreshold BN, BED, ED not otherwise specified or subthreshold ED, and full-syndrome BN and BED.

### Implications

The results from this study suggest that the guided web-based intervention SB-AN is the first indicated prevention program to significantly reduce risk factors and symptom progression of AN symptoms such as restrained eating and low body weight. The intervention also shows promise for late onset of newly emerging full-syndrome and subclinical AN syndromes. Compared with studies reporting on ED onset, this study clearly succeeded in recruiting women with low or lower BMI and at higher risk for AN than participants of previous prevention trials. Consequently, the study yielded the highest rates of newly emerging subclinical and clinical AN. Although full-syndrome DSM-IV ED cases, including AN, were excluded from the study because of the revision of the weight criterion with the introduction of DSM-5, a relatively high rate (21/168, 12.5%) of women fulfilling criteria for full-syndrome and subclinical AN were unintentionally included in the study. For these women, the intervention proved beneficial in reducing symptom progression. The intervention could therefore be recommended as a specific intervention for women with low weight and high restrained eating to clinicians and health care providers who often underestimate these risks. However, the recruitment process also demonstrates that women at risk for AN are not easily enrollable in an intervention targeting low body weight, resulting in a considerable proportion (128/863, 14.8%) of participants screening positive who declined participation [53].

### Conclusions

Although SB-AN overall proved moderately effective for women at risk for AN, future studies should try to improve the reach and uptake of the intervention, that is, examine its effects in more diverse populations; try to further increase motivation to change, especially in participants who are underweight; or examine whether reach can be increased by use of a mobile-based version of the intervention. Moderator variables—part of a separate analysis—might also shed more light on how to better tailor the intervention to increase reach and effectiveness for specific subgroups of participants. The consideration of health economic outcomes could demonstrate further benefits of the intervention compared with the costs for treating medical complications and treatment of symptoms of AN and subclinical AN.

## Appendix

Multimedia Appendix 1 Secondary outcomes.

Multimedia Appendix 2 CONSORT-eHEALTH checklist (V 1.6.1).

## Abbreviations

AN: anorexia nervosa
ARR: absolute risk reduction
BED: binge eating disorder
BN: bulimia nervosa
CG: control group
DSM: Diagnostic and Statistical Manual of Mental Disorders
ED: eating disorder
EDI: Eating Disorder Inventory
FU: follow-up
GLMM: generalized linear mixed model
IG: intervention group
KKS: Koordinierungszentrum für Klinische Studien
LOCF: last observation carried forward
NNT: number needed to treat
SB-AN: Student Bodies-Anorexia Nervosa
WCS: Weight Concerns Scale

## Data Availability

Participant level data are available on request from the corresponding author. Participants’ consent on data sharing was not obtained, but the presented data are anonymized and risk of identification is low.
