# Supplementary material for: Indicated Web-Based Prevention for Women With Anorexia Nervosa Symptoms: Randomized Controlled Efficacy Trial
Source: J Med Internet Res. 2022 Jun 2;24(6):e35947. doi: 10.2196/35947 (PMC9204567; doi:10.2196/35947)
Supplement: Multimedia Appendix 1 [file jmir_v24i6e35947_app1.docx]

Secondary outcomes (N=168).

|  | | | | | | | Intervention group (n=84) | | | | | | | | | | | | Control group (n=84) | | | | Adjusted mean difference (95% CI), after the intervention | *P* value | Adjusted mean difference (95% CI), FU6^a^ | *P* value | Adjusted mean difference (95% CI), FU12^b^ | *P* value |
| --- | --- | --- | --- | --- | --- | --- | --- | --- | --- | --- | --- | --- | --- | --- | --- | --- | --- | --- | --- | --- | --- | --- | --- | --- | --- | --- | --- | --- |
|  | | | | | | | Baseline, mean (SD) | | | | | | | | | After the intervention, mean (SD) | FU6, mean (SD) | FU12, mean (SD) | Baseline, mean (SD) | After the intervention, mean (SD) | FU6, mean (SD) | FU12, mean (SD) |  |  |  |  |  |  |
|  |  |  |  |  |  |  | |  |  |  |  |  |  |  |  |  |  |  |  |  |  |  |  |  |  |  |  |  |
| BDI-II^c^ | | | | | | | 11.74 (9.12) | | | | | | | | | 6.65 (8.51) | 7.31 (8.36) | 10.35 (8.37) | 13.26 (9.12) | 10.95 (8.66) | 11.06 (8.74) | 10.35 (8.37) | 0.4 (0.06 to 0.75) | .02 | 0.32 (–0.02 to 0.67) | .07 | 0.36 (–0.01 to 0.73) | .06 |
| BMI (UW^d^-T0^e^)^f^ | | | | | | | 18.43 (0.89) | | | | | | | | | 19.35 (0.86) | 19.52 (0.86) | 18.97 (0.85) | 18.38 (0.89) | 18.86 (0.87) | 18.89 (0.86) | 18.97 (0.85) | 0.48 (–0.09 to 1.05] | .10 | 0.64 (0.05 to 1.22) | .03 | 0.67 (0.02 to 1.31) | .04 |
| BSI^g^ | | | | | | | 0.65 (0.54) | | | | | | | | | 0.43 (0.5) | 0.41 (0.49) | 0.60 (0.49) | 0.78 (0.54) | 0.62 (0.51) | 0.66 (0.51) | 0.60 (0.49) | 0.17 (–0.17 to 0.51) | .36 | 0.28 (–0.06 to 0.63) | .11 | 0.2 (–0.17 to 0.56) | .29 |
| CIA^h^ total score | | | | | | | 12.85 (12.14) | | | | | | | | | 6.67 (6.2) | 6.54 (6.08) | 9.61 (8.98) | 16.19 (15.34) | 12.28 (11.5) | 11.18 (10.81) | 9.61 (8.98) | 0.44 (0.1 to 0.79) | .01 | 0.31 (–0.03 to 0.66) | .08 | 0.24 (–0.12 to 0.61) | .19 |
| EDE^i^ total score | | | | | | | 2.21 (1.03) | | | | | | | | | 1.45 (0.98) | 1.31 (0.97) | 1.58 (0.96) | 2.49 (1.03) | 2.04 (1.00) | 1.79 (0.98) | 1.58 (0.96) | 0.34 (>0.00 to 0.68) | .05 | 0.22 (–0.13 to 0.57) | .21 | 0.17 (–0.19 to 0.54) | .36 |
| EDE WC^j^ | | | | | | | 2.12 (1.47) | | | | | | | | | 1.43 (0.95) | 1.27 (0.84) | 1.60 (1.04) | 2.49 (1.72) | 1.95 (1.31) | 1.82 (1.21) | 1.60 (1.04) | 0.22 (–0.11 to 0.56) | .19 | 0.31 (–0.04 to 0.66) | .08 | 0.25 (–0.12 to 0.61) | .18 |
| EDE EC^k^ | | | | | | | 1.08 (1.17) | | | | | | | | | 0.73 (0.92) | 0.59 (0.8) | 0.63 (0.85) | 1.43 (1.41) | 1.04 (1.15) | 0.81 (0.98) | 0.63 (0.85) | 0.05 (–0.28 to 0.39) | .75 | 0.03 (–0.31 to 0.38) | .85 | 0.05 (–0.31 to 0.42) | .78 |
| EDE RS^l^ | | | | | | | 2.49 (1.82) | | | | | | | | | 1.22 (0.89) | 1.19 (0.94) | 1.40 (1.04) | 2.37 (1.7) | 1.93 (1.4) | 1.51 (1.1) | 1.40 (1.04) | 0.7 (0.35 to 1.04) | <.001 | 0.38 (0.02 to 0.72) | .04 | 0.56 (0.19 to 0.93) | .003 |
| EDE SC^m^ | | | | | | | 2.90 (1.24) | | | | | | | | | 2.03 (1.18) | 1.87 (1.17) | 2.32 (1.16) | 3.15 (1.24) | 2.75 (1.21) | 2.53 (1.19) | 2.32 (1.16) | 0.44 (0.1 to 0.78) | .01 | 0.39 (0.03 to 0.74) | .03 | 0.32 (–0.04 to 0.69) | .08 |
| EDI2^n^ BD^o^ | | | | | | | 36.98 (10.04) | | | | | | | | | 31.55 (9.35) | 31.85 (9.18) | 35.51 (9.2) | 39.61 (10.04) | 37.57 (9.53) | 36.21 (9.62) | 35.51 (9.2) | 0.46 (0.11 to 0.8) | .009 | 0.23 (–0.11 to 0.58) | .18 | 0.37 (0.01 to 0.74) | .05 |
| EDI2 DFT^p^ | | | | | | | 26.92 (7.97) | | | | | | | | | 21.25 (7.47) | 20.87 (7.34) | 24.57 (7.35) | 29.06 (7.97) | 28.08 (7.59) | 24.94 (7.66) | 24.57 (7.35) | 0.75 (0.4 to 1.1) | <.001 | 0.31 (–0.04 to 0.66) | .08 | 0.45 (0.08 to 0.82) | .02 |
| WCS^q^ | | | | | | | 55.85 (18.09) | | | | | | | | | 45.37 (16.99) | 42.31 (16.7) | 51.56 (16.72) | 63.16 (18.09) | 59.97 (17.25) | 53.93 (17.41) | 51.56 (16.72) | 0.51 (0.16 to 0.85) | .004 | 0.3 (–0.04 to 0.65) | .09 | 0.27 (–0.1 to 0.64) | .15 |
| Knowledge test | | | | | | | 18.11 (2.45) | | | | | | | | | 21.25 (2.33) | 20.79 (2.29) | 20.14 (2.29) | 18.02 (2.45) | 19.4 (2.35) | 19.63 (2.37) | 20.14 (2.29) | 0.83 (0.47 to 1.18) | <.001 | 0.51 (0.16 to 0.86) | .004 | 0.23 (–0.14 to 0.59) | .22 |
| Binge eating episodes^r^ | | | | | | | 6.16 (4.95) | | | | | | | | | 2.61 (2.17) | 0.64 (0.78) | 1.49 (1.41) | 5.87 (4.73) | 2.94 (2.43) | 1.19 (1.21) | 1.49 (1.41) | 0.26 (–0.38 to 0.89) | .43 | 0.64 (–0.06 to 1.34) | .07 | 1.03 (0.18 to 1.86) | .01 |

^a^FU6: 6-month follow-up.

^b^FU12: 12-month follow-up.

^c^BDI-II: Beck Depression Inventory-2.

^d^UW: underweight.

^e^T0: screening.

^f^Intervention group, n=31; control group, n=31.

^g^BSI: Brief Symptom Inventory.

^h^CIA: Clinical Impairment Assessment.

^i^EDE: Eating Disorder Examination.

^j^WC: Weight Concern.

^k^EC: Eating Concern.

^l^RS: Restraint.

^m^SC: Shape Concern.

^n^EDI-2: Eating Disorder Inventory-2.

^o^BD: Body Dissatisfaction.

^p^DFT: Drive for Thinness.

^q^WCS: Weight Concerns Scale.

^r^Intervention group, n=26; control group, n=28.
